# Thermal-porosity characterization data of additively manufactured Ti–6Al–4V thin-walled structure via laser engineered net shaping

**DOI:** 10.1016/j.dib.2023.109722

**Published:** 2023-10-24

**Authors:** Christian Zamiela, Wenmeng Tian, Shenghan Guo, Linkan Bian

**Affiliations:** a Department of Industrial and Systems Engineering, Mississippi State University, Mississippi State, MS 39762, United States; b Center for Advanced Vehicular Systems (CAVS), Mississippi State University, Mississippi State, MS 39762, United States; c School of Manufacturing Systems and Networks, Arizona State University, Mesa, AZ 85296, United States

**Keywords:** Additive manufacturing, Laser engineering net shaping, Melt pool, Process monitoring, Pyrometer thermal imaging, X-ray computed tomography

## Abstract

In-process thermal melt pool images and post-fabrication porosity labels are acquired for Ti-6Al-4V thin-walled structure fabricated with OPTOMEC Laser Engineered Net Shaping (LENS™) 750 system. The data is collected for nondestructive thermal characterization of direct laser deposition (DLD) build. More specifically, a Stratonics dual-wavelength pyrometer captures a top-down view of the melt pool of the deposition heat-affected zone (HAZ), which is above $1000^{\circ }C$, and Nikon X-Ray Computed Tomography (XCT) XT H225 captures internal porosity reflective of lack of fusion during the fabrication process. The pyrometer images provided in Comma Separated Values (CSV) format are cropped to center the melt pool to temperatures above 1000℃, indicative of the shape and distribution of temperature values. Melt pool coordinates are determined using pyrometer specifications and thin wall build parameters. XCT porosity labels of sizes between 0.05 mm to 1.00 mm are registered within 0.5 mm of the melt pool image coordinate. An XCT porosity-labeled table provided in the Excel spreadsheet format contains time stamps, melt pool coordinates, melt pool eccentricity, peak temperature, peak temperature coordinates, pore size, and pore label. Thermal-porosity data utilization aids in generating data-driven quality control models for manufacturing parts anomaly detection.

Specifications Table

**Table:** 

Subject	Data Mining and Statistical Analysis, Mechanical Engineering, Industrial Engineering
Specific subject area	Porosity, Thermal Monitoring, Abnormality Detection, Laser-Based Additive Manufacturing
Data format	Raw, Filtered
Type of data	Image, Table
Data collection	**Thermal Monitoring:** An OPTOMEC LENS^TM^ 750 system additively manufactured thin-walled structure via Laser Engineered Net Shaping (LENS) using Ti-6AL-4V powder. A Stratonics dual-wavelength pyrometer monitors the thermal response of the melt pool during the manufacturing process. **Porosity Labels:** X-Ray data is collected post-fabrication of thin-walled structures with a Nikon X-Ray Computed Tomography (XCT) XT H225 equipped with an X-Ray beam tube, a high-voltage generator, a sample manipulator, and a panel detector. MyVGL Studio MAX DefX porosity algorithm calculates the location and volume of porosity within the XCT three-dimensional tensor.
Data source location	Center for Advanced Vehicular Systems (CAVS), Starkville, Mississippi 39759, USA
Data accessibility	Thermal-Porosity Dataset: Repository name: Harvard Dataverse Direct URL to data: https://dataverse.harvard.edu/dataset.xhtml?persistentId=doi:10.7910/DVN/BWHYEH

## 1 Value of the Data

• Data registration of pyrometer melt pool data and XCT provides the ability to characterize the metal fusion process.
• Data indicative of porosity is urgently needed for component certification due to the high process uncertainty in metal-based AM processes.
• Data provides the ability to develop data-driven analytical models for abnormality detection to further insights into quality control for laser-based additive manufacturing processes.
• Data allows the analysis of the deposition melt pool characteristics to post-fabrication porosity quality measures.
• Data allows for investigating the relationship between processing parameters, in-situ data characteristics, and the complex pore formation and solidification process.
• Data supports the training of machine learning (ML) and statistical surrogate models for laser-based additive manufacturing processes.

## 2 Data Description

**Fig. 1:**
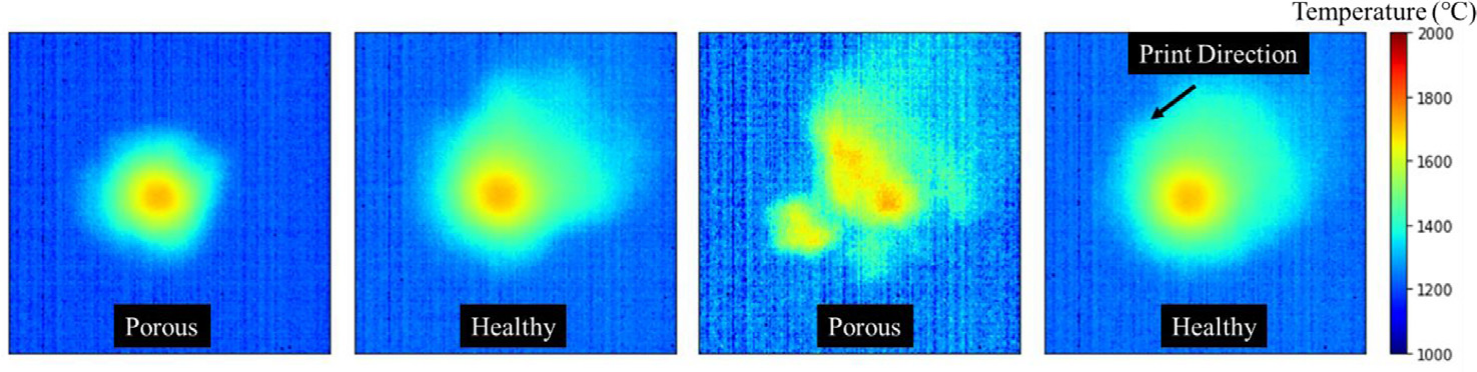
Cropped melt pool images with print direction visual and porosity labels.

The data presented here consists of a folder with 1,564 cropped pyrometer melt pool images in CSV format and an thermal-porosity table in Excel format. The cropped melt pool images are visualized in Fig. 1. Each pyrometer melt pool CSV displays a matrix of temperature values in degrees Celsius that correspond to the *Frame* within thermal-porosity Excel sheet. Table 1 is a few observations of porosity-labeled excel sheet contains the following columns describing melt pool images and linking porosity:

1. *Frame*: The corresponding pyrometer melt pool CSV label.
2. *Time (Sec)*: The time stamp of captured pyrometer melt pool CSV.
3. *Y (mm)*: The coordinate value of pyrometer melt pool CSV in y-direction.
4. *Z (mm)*: The coordinate value of pyrometer melt pool CSV in z-direction.
5. *MP Area*: The area of pyrometer melt pool CSV above 1660°C.
6. *MP Eccentricity*: The eccentricity of the pyrometer melt pool CSV.
7. *Tmax*: The maximum temperature in pyrometer melt pool CSV.
8. *Row*: The pyrometer melt pool CSV row location of maximum temperature.
9. *Column*: The pyrometer melt pool CSV column location of maximum temperature.
10. *Porosity Label*: The porosity label linked to pyrometer melt pool CSV.
11. *Porosity Size (mm)*: The porosity size linked to pyrometer melt pool CSV.

**Table 1:** Thermal-porosity table.

Frame	Time (Sec)	Y (mm)	Z (mm)	Layer	MP Area	MP Eccentricity	Tmax	Row	Column	Porosity Label	Porosity Size (mm)
1	0	0	0	1	2745	0.197	1875	98	96	1	0.419
2	0.152	1.936	0	1	1106	0.351	1725	96	87	1	0.332
3	0.305	3.872	0	1	1318	0.434	1739	100	85	1	0.342
4	0.457	5.808	0	1	1572	0.424	1756	95	91	1	0.338
5	0.610	7.744	0	1	1444	0.374	1749	101	87	1	0.345
6	0.762	9.68	0	1	945	0.276	1711	91	87	1	0.282
7	0.915	11.62	0	1	1744	0.287	1763	101	94	0	0
⋮	⋮	⋮	⋮	⋮	⋮	⋮	⋮	⋮	⋮	⋮	⋮

The thin wall builds coordinates *Y (mm)* and *Z (mm)* within thermal-porosity Excel sheet are linked to XCT porosity coordinates within 0.5 mm range. The *Time (Sec)* is based on the Frame Rate of Pyrometer. The x-coordinate is not present in thermal-porosity Excel sheet because it is constant due to moving in one direction along the y-axis. Moreover, the melt pool images are characterized by *Porosity Labels* along with *Porosity Size (mm)* seen in Table 2. *MP Area, MP Eccentricity, Tmax, Row,* and *Column* provide descriptive characteristics of the heat-affected zone (HAZ) and shape of the melt pool for melt fusion.

**Table 2:** Summary of pyrometer CSVs, XCT porosity labels, and build parameter data description.

Parameters	Summary Values
**Pyrometer CSVs**	
Array size	200×200
Pixel pitch	6.45μm
Temperature Range	1000-2500°C
Frame Rate	∼ 6.67 fps
Eccentricity Range	0.13−0.95mm
Maximum Temperature Range	1602-2107°C
Melt Pool Area Range	0−7274pixels
Number of CSVs Per Layer Range	16-34 layers
Average Signal-to-Noise Ratio (SNR)	10.97
**XCT Porosity Labels**	
Binary Labels	0 = No Porosity, 1 = Porosity
Number of Label Occurrences	0 = 1490, 1 = 70
Porosity Size Range	0.05−0.98mm
Image Class Correlation	0.78
**Thin Wall Build Parameters**	
Build Layers	60
Print Direction	Unidirectional
Print Speed	12.7mm/s
Layer Thickness	0.508mm

Table 2 provides a summary of parameters within the dataset of Pyrometer CSV's, XCT Porosity Labels, and Thin Wall Build Parameters. Fig. 1 provides examples of melt pool image CSVs. Note the shape and print direction can be visualized in 200×200 resolution images. The melt pool CSVs provide in-process traits of the metal fusion process, while the porosity provides ground truth fusion quality between layers post-fabrication. The files can be found on Harvard Dataverse Repository: https://dataverse.harvard.edu/dataset.xhtml?persistentId=doi:10.7910/DVN/BWHYEH.

## 3 Experimental Design, Materials and Methods

**Fig. 2:**
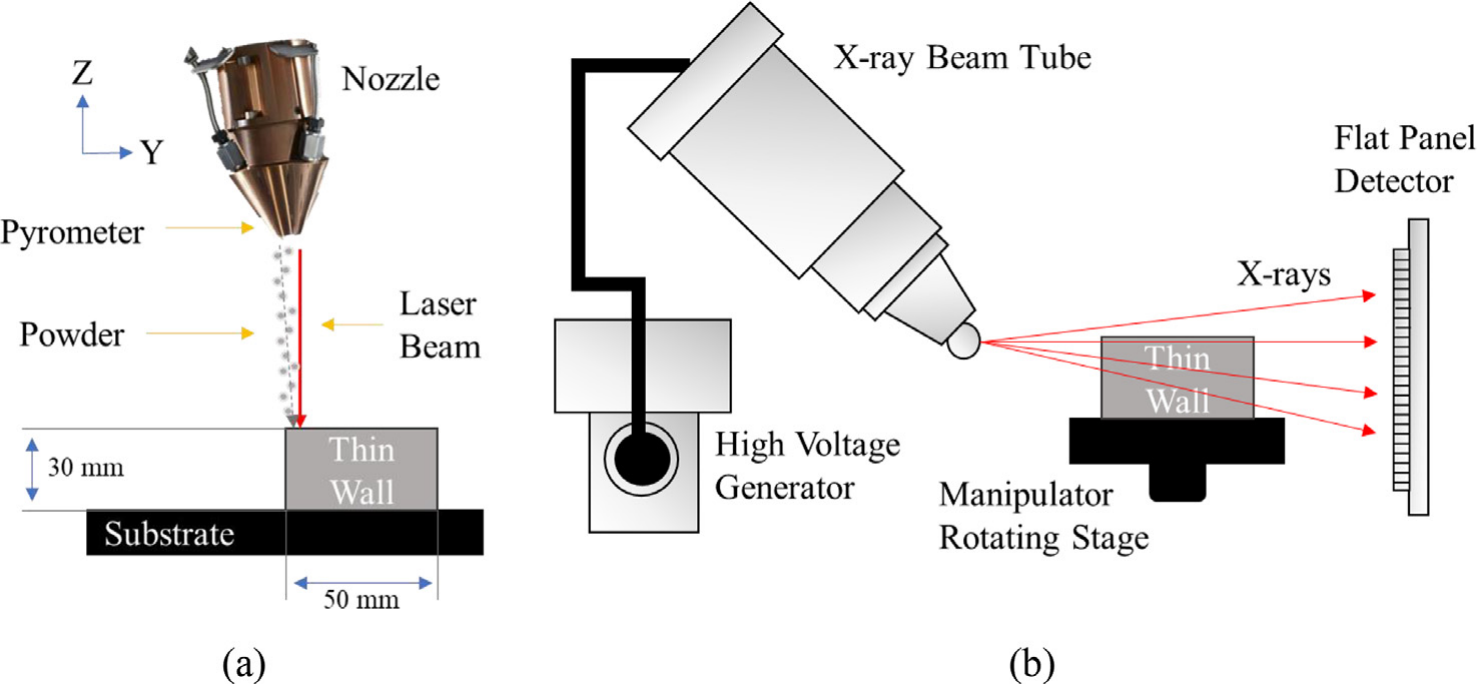
Data collection setup. (a) LENS build chamber and sensor setup. (b) XCT sensor setup.

An OPTOMEC LENS 750 system additively manufactures a thin wall with Ti-6AL-4V powder. The raw pyrometer images, manufacturing parameters, and thermal data collection details derive from [1], and the data capturing, processing, and registration procedure is described in this section and visualized in Fig. 2a. A Stratonics Dual Wavelength Pyrometer captures an aerial perspective through the nozzle of the melt pool at the laser deposition location indicative heat affected zone (HAZ) where layer fusion is taking place [14]. The 752×480resolution pyrometer images are cropped to 200×200 resolution centered around the melt pool above $1000^{\circ }C$ and within visible spectrum of pyrometer $1000-2500^{\circ }C.\;$The pyrometer threshold temperature is ideal for data collection due to metal phase change temperatures during printing. For instance, the melting point (liquidus phase) of Ti-6Al-4V powder is $1660^{\circ }C$, and the beta transus (metallurgy phase change) temperature is 995°C [15].

X-ray porosity records are captured via Nikon X-Ray Computed Tomography XT H225 [16] and processed with VGStudio Max DefX porosity algorithm [17]. The thin wall attached to a substrate is placed onto a rotating stage. An X-ray beam tube with an approximate voltage of 180 kV and a current of 98 μA projects a full 3D cone of X-rays with an approximate wavelength of 0.1033 angstroms onto a 2-D flat panel detector to capture structural data on the ionized matter. This cone beam scanner irradiates the whole object throughout the 360° scan to obtain 3D data for the entire thin wall (Fig. 2b). The DefX porosity algorithm within VGStudio processes XCT data to detect pores with sizes ranging from 0.05 mm to 1.00 mm because pores with a diameter of less than 0.05 mm are difficult to identify. Pyrometer and X-Ray porosity record data registration is performed by first utilizing the thin wall build parameters (print direction, speed, and layer thickness) and pyrometer frame rate to determine the melt pool's location throughout time. The coordinate of each pyrometer CSV melt pool is manually matched to pores that are within a 0.5 mm range of melt pool images. The combined images and XCT labeling produce an image roughly every 0.29 seconds, covering an estimated distance of about 1.936 millimeters between images.

This dataset has been adapted and utilized in several works on data-driven thermal monitoring for porosity detection [2], [3], [4], [5], [6], [7], [8], [9], [10], [11], online transfer learning [12], and surrogate modeling [13]. These works aim to address quality assurance challenges by developing methods to measure statistical changes in melt pool data and characterizing relationships between thermal responses and porosity labels. In addition, feature extraction techniques and supervised learning methods are developed in these works to showcase the use of *in-situ* sensing for porosity detection. The advantages of producing these works are developing in-process monitoring methods for rapid porosity detection and automating the feature extraction process. Furthermore, expanding the supervised learning model to an online and transfer learning model improves real-time model adaptability and ML transferability for incoming data from new part geometries to predict porosity. This capability is beneficial when the historical data are too scarce to train the ML algorithms sufficiently. Lastly, a thermal surrogate modeling approach to emulate population profiles of the transient thermal signatures. Generating images for the thermal dynamics of the melt pool conditionally of the deposition layer images is necessary for balancing datasets and enhancing model training for characterizing the inherited relationship.

## 4 Limitations

The data collection and registration with metadata pose the following limitations and uncertainty. Firstly, pyrometer CSV temperature values depend upon the material's emissivity and surface quality. Changes in the surface properties due to powder distribution could affect the emissivity and hence the recorded temperature values. Secondly, the Optomec LENS 750 system prints 12.7 mm/s, and the Stratonics pyrometer captures melt pool images at a rate of 6.7 fps causing missing melt pool images within each layer. Moreover, coordinate positional alignment of pyrometers CSVs to build geometry is based on build parameters resulting in some uncertainty of melt pool location. Lastly, matching of pyrometer CSV melt pool geometry to the observed XCT geometry presents variability. Missing and sparsely distributed pyrometer CSV data presents challenges in aligning porosity labels that could limit data-driven model pattern detection.

## Ethical Statement

The authors comply with the ethical guidelines of the journal. Humans, animals, or data from social media are not involved in this research.

## CRediT authorship contribution statement

**Christian Zamiela:** Data curation, Writing – original draft. **Wenmeng Tian:** Writing – review & editing. **Shenghan Guo:** Writing – review & editing. **Linkan Bian:** Supervision, Funding acquisition.

## Data Availability

Thermal-Porosity Characterization Data of Additively Manufactured Ti–6Al–4V Thin-walled Structure via Laser Engineered Net Shaping (Original data) (Dataverse)
